# Abdominal Epilepsy Masked with Hiccups in a Patient with Intracranial Malignant Glioma

**DOI:** 10.7759/cureus.6338

**Published:** 2019-12-10

**Authors:** Salman Assad, Varun Dobariya, Mehr Zahid, Shuja A Malik

**Affiliations:** 1 Internal Medicine, Marshall University School of Medicine, Huntington, USA; 2 Internal Medicine, University of Lahore, Lahore, PAK; 3 Internal Medicine, Nawaz Sharif Medical College, University of Gujrat, Gujrat, PAK

**Keywords:** hiccups, epilepsy, seizure

## Abstract

Abdominal hiccups are often masked by abdominal epilepsy (AE) in clinical settings. Spontaneous arrhythmic muscular movements sometimes raise the suspicion for abdominal myoclonus as well. AE is an atypical and rare cause of seizure disorder. It is a manifestation of different transient abdominal complaints correlating with abnormal electroencephalogram (EEG) changes and adequate response to anti-epileptic drugs. We present a case of an 80-year-old female who presented with an episode of tonic-clonic seizure that lasted for almost 10 minutes. The patient was confused and had a facial droop. She had another episode of seizure with a perseverative speech followed by left facial drooping and left upper extremity weakness. She continued having fluctuating mental status and left-sided hemiparesis with intermittent abdominal twitching. She was getting more bradykinetic than bradyphrenic. The computed tomography (CT) scan of the head and magnetic resonance imaging (MRI) of the brain showed a parietal lobe mass that was confirmed on biopsy as malignant glioma. The long-term video monitoring EEG report showed the occurrence of persistent right parietal spikes with background slowing. The brain mass was later treated with radiation therapy and surgery.

## Introduction

Abdominal epilepsy (AE) is an uncommon and undocumented cause of parietal lobe tumor in the literature. It is characterized by diverse abdominal complaints, electroencephalogram (EEG) abnormalities, and favorable response to anti-epileptic drugs. Often, the abdominal epileptiform movements are masked by abdominal hiccups. Focal epilepsy with generalization can present with diversified patterns [1]. 

## Case presentation

**Figure 1:**
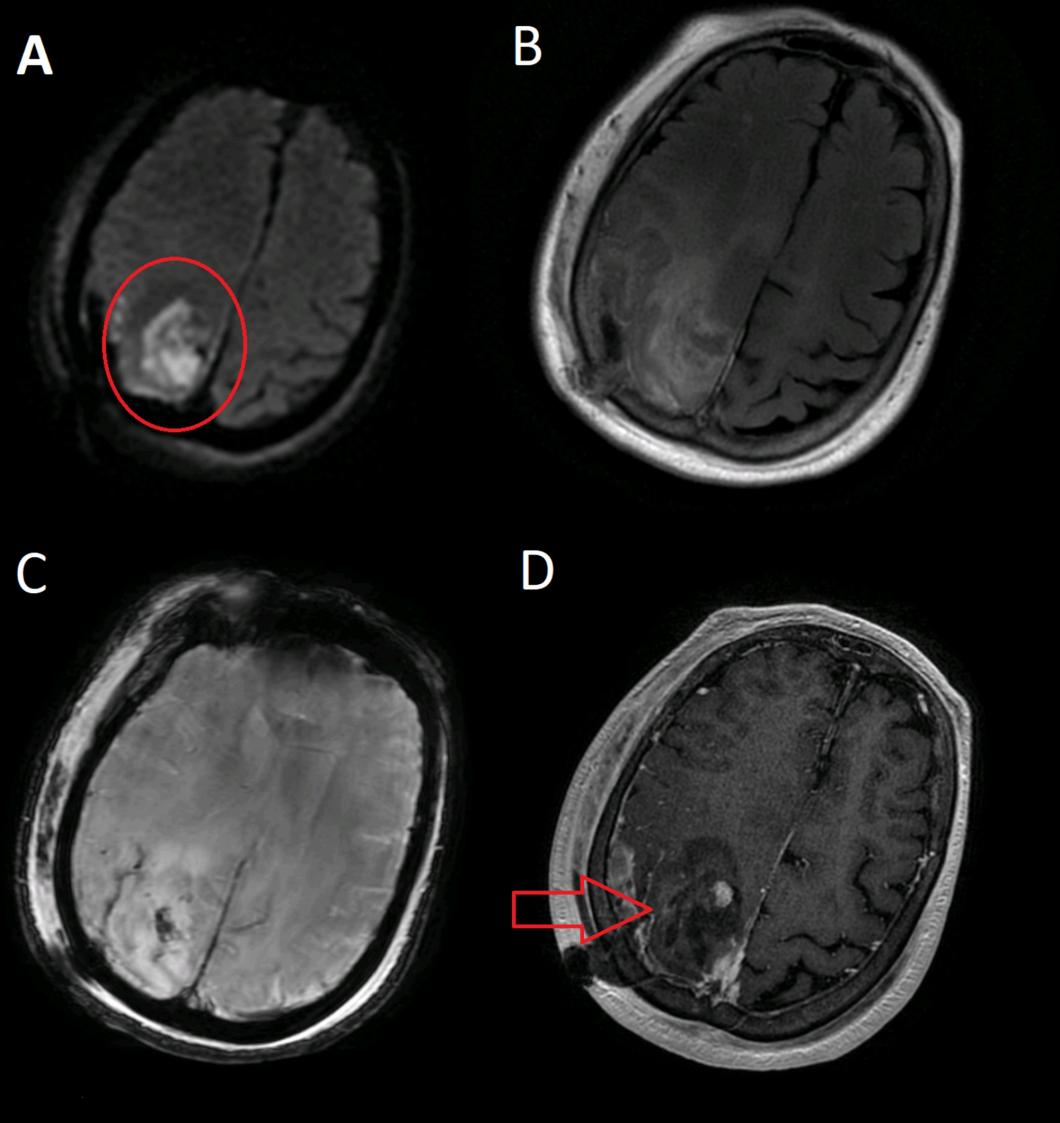
MRI of the brain with and without contrast (A-D) Right parieto-occipital glioma with defined margins (red circle and arrow)

We present a case of an 80-year-old female with a history of left breast cancer status post-lumpectomy who presented with an episode of a tonic-clonic seizure that lasted for 10 minutes. In the past, she had an early-stage hormone receptor (HR)-positive/ human epidermal growth factor receptor 2 (HER2)-negative that was treated with surgery followed by radiation. During hospitalization, she had another episode of seizure with a perseverative speech followed by left facial droop and left upper extremity weakness. She continued having fluctuating mental status with left-sided hemiparesis and intermittent abdominal twitching. During this epileptic episode, which lasted over five minutes, there was no involvement of the face, arm, or leg. She did not lose consciousness but was getting more bradykinetic than bradyphrenic, with left-sided body weakness. It looked like a hiccup initially but had the spontaneous rhythmic movements localized to the abdomen. Clinically, it appeared likely to be an epileptic event with atypical features. Complete blood count and metabolic workup were unremarkable. Computed tomography (CT) scan of the head and magnetic resonance imaging (MRI) of the brain showed a right parietal lobe mass with an estimated size of 3.9 x 2.1 x 3 cm with surrounding vasogenic edema (Figure 1).

**Figure 2:**
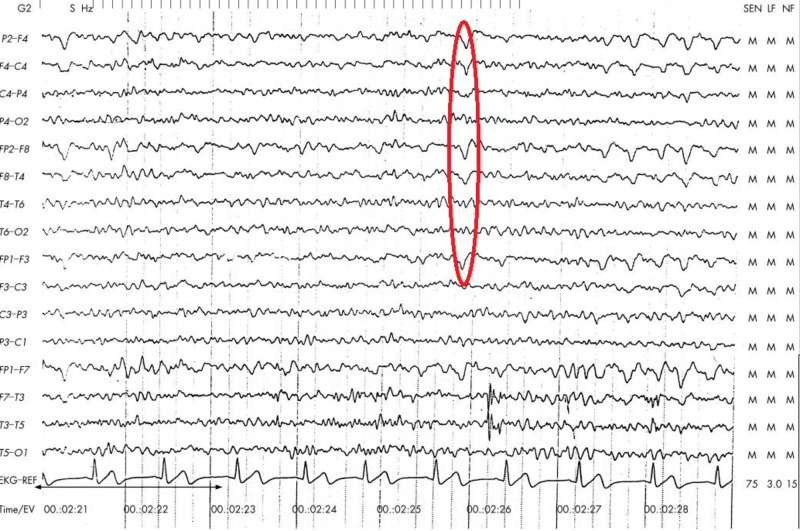
Electroencephalogram showing right temporo-parietal spikes (circled)

The long-term video monitoring EEG report showed the occurrence of persistent right parietal spikes with background slowing. There was a spread of interictal epileptiform discharges to the right frontal and temporal regions. Findings are consistent with a focal area that is potentially epileptogenic (Figure 2).

The patient was initiated on lorazepam 4 mg intravenous (IV) once a day, fosphenytoin 1,300 mg IV once a day, and dexamethasone 4 mg IV once a day followed by levetiracetam 500 mg oral tablet twice a day to prevent further episodes of seizures. She had been taking anastrozole 1 mg oral daily tablet after left breast lumpectomy. The craniotomy was done to remove the lesion, followed by stereotactic radiation. Later, the result of a brain biopsy confirmed it as malignant glioma. However, her abdominal epileptic episodes were controlled after increasing the dose of levetiracetam to 750 mg twice a day.

## Discussion

AE is a rare seizure disorder of episodic gastrointestinal manifestations with ECG abnormalities. The diagnosis of AE is a challenge, and its prevalence is unclear [1]. In a small pediatric case series, its prevalence was observed to be higher in females, with a male-to-female ratio of 1:2, indicating gender predilection. AE is also known as autonomic epilepsy, which is characterized by paroxysmal episodic abdominal and peri-umbilical pain due to central nervous system disturbance, with an abnormal EEG. AE is usually diagnosed late because of non-specific intermittent symptoms [2]. Yunus et al. reported a case of a five-year-old pediatric patient with recurrent paroxysmal abdominal pains for the last seven to eight months. The child had sudden attacks lasting one to two minutes with palpitation and stuttering every two to three days that resolved spontaneously. The only positive finding was the EEG abnormality during the abdominal pain, which showed a generalized spike precipitated by hyperventilation. The patient had an excellent response to anti-convulsants, with a normal EEG after one month [3].

The key to diagnosing AE is a high clinical suspicion with the abnormal abdominal movements and a concurrent ECG irregularity. AE responds successfully to a single or double anti-epileptic agent. Kraimer et al. reported a case of a 16-year-old male with symptoms from two years of age, consisting of abdominal pain and distension lasting for several hours resolving spontaneously with rare epileptiform discharges over the right frontal region [3]. The seizure was refractory to multidrug therapy, and the patient underwent vagal nerve stimulation therapy, which reduced the incidence of seizures, and the anti-epileptic drugs were gradually withdrawn [4]. Though most of the cases reported are of the patients belonging to pediatric age groups, we presented an example of an elderly lady with tonic-clonic seizure associated with hemiparesis. Our patient had a seizure that lasted for five minutes without any involvement of the face, arm, or loss of consciousness but had a rhythmic focal abnormal movement on the left side of the abdomen. On further investigation, the patient was found to have a mass in the right parietal region that was treated with surgery followed by radiation therapy. After a craniotomy, the episodes of abdominal seizures were controlled with long-term anti-epileptic drug treatment.

## Conclusions

AE has been frequently misdiagnosed as abdominal hiccups in clinical settings and should be correlated with EEG findings to rule out any epileptogenic event. This emphasizes the need for medical personnel to consider AE in a clinical context with an underlying malignant brain lesion.

## References

[1] Harshe DJ, Harshe SD, Harshe GR, Harshe GG (2016). Abdominal epilepsy in an adult: a diagnosis often missed. J Clin Diagn Res.

[2] Dutta SR, Hazarika I, Chakravarty BP (2007). Abdominal epilepsy, an uncommon cause of recurrent abdominal pain: a brief report. Gut.

[3] Kraimer KL, Kochanski RB, Lynn F, Smith M, Sani S (2019). Abdominal epilepsy treated with vagal nerve stimulation: a case report. Oper Neurosurg (Hagerstown).

[4] Yunus Y, Sefer U, Dondu UU, Ismail O, Yusuf E (2016). Abdominal epilepsy as an unusual cause of abdominal pain: a case report. Afr Health Sci.

